# Case Report: An Unusual Case of Ectopic ACTH Syndrome Caused by Mediastinal Paraganglioma

**DOI:** 10.3389/fendo.2021.790975

**Published:** 2022-01-05

**Authors:** Bo Li, Zhe Yan, Hui Huang

**Affiliations:** Department of Endocrinology and Metabolism, West China Hospital, Sichuan University, Chengdu, China

**Keywords:** ectopic ACTH syndrome, Cushing’s syndrome, paraganglioma, mediastinal, PET/CT

## Abstract

Ectopic adrenocorticotrophic hormone (ACTH) syndrome is not common, which is more unusual when caused by paraganglioma. We herein present a 40-year-old Chinese male who was diagnosed with ACTH-dependent Cushing’s syndrome. However, the localization of the ACTH source was troublesome due to the inconsistent results of the high-dose dexamethasone suppression test and the desmopressin stimulation test. Bilateral inferior petrosal sinus sampling was performed, and ectopic ACTH syndrome was diagnosed. After ^68^Ga-DOTATATE-PET/CT and ^18^F-FDG-PET/CT were performed, it was localized in the anterior mediastinum. Post-operation histopathology demonstrated an ACTH-secreting mediastinal paraganglioma. The patient obtained complete clinical remission after a mediastinal tumorectomy.

## Introduction

Cushing’s syndrome (CS) is a general term for diseases caused by excessive cortisol secretion in the adrenal cortex due to various reasons. The accompanying clinical presentations are all attributed to hypercortisolism. Ectopic adrenocorticotrophic hormone (ACTH) syndrome, a rare type of ACTH-dependent CS, is a disease referable to the abnormal secretion of ACTH from tumors outside of the pituitary, which stimulates bilateral adrenal gland hyperplasia and excessive secretion of cortisol. According to the literature, the most common cause of ectopic ACTH syndrome (EAS) is lung cancer (1). Paraganglioma-associated ACTH-secreting tumor was rarely reported in clinics to date (2).

Here we report a Chinese patient diagnosed with ACTH-dependent CS, which was ultimately attributed to an ACTH-secreting mediastinal paraganglioma.

## Case Presentation

A 40-year-old male complaining of pitting edema in the whole body with limb weakness, polydipsia, and polyuria was admitted to our Endocrinology Unit. No one in his family suffers from a similar disease.

The physical examinations revealed vital signs of heart rate of 91 beats/min and blood pressure of 148/84 mmHg. He has abdominal obesity with a BMI of 29.5 kg/m^2^ and a waist–hip ratio of 0.98. The patient had a classic Cushing’s syndrome appearance, presented as central obesity, moon face, buffalo back, scattered bruises in the upper limbs, and pitting edema of the whole body.

**Figure 1:**
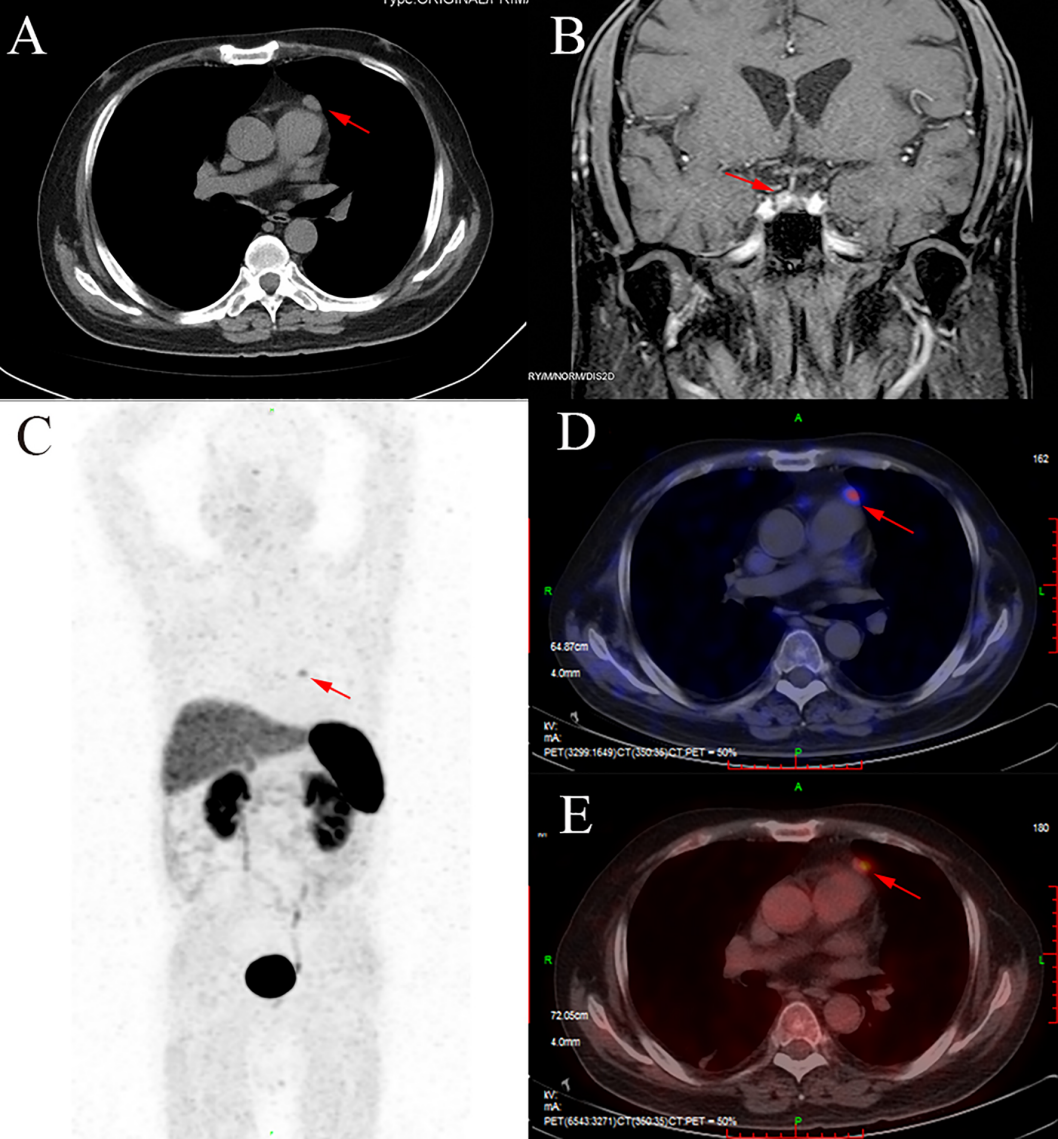
The CT, MRI, and PET/CT images of this patient. **(A)** A soft tissue density nodule in the mediastinum (red arrow). **(B)** A nodule in the right side of the pituitary (red arrow). **(C, D)** The anterior mediastinal nodule with increased ^68^Ga uptake (red arrow). **(E)** The anterior mediastinal nodule with increased fluorodeoxyglucose uptake (red arrow).

The laboratory examination showed an increased level of 24-h urine-free cortisol (24-h UFC) and the disappearance of circadian rhythm in plasma total cortisol (PTC), accompanied by an elevated ACTH level, which confirmed the ACTH-dependent Cushing’s syndrome diagnosis for this patient. These were also presented with severe hypokalemia, hyperglycemia, and alkalosis, which were relevant to CS. The high-dose dexamethasone suppression test (HDDST) showed a low suppressing rate of plasma ACTH and PTC. However, the 24-h UFC was suppressed by more than 50%. Desmopressin stimulation test was performed, and the results showed an intensively stimulated rate of ACTH level, with a peak rate 836% compared to baseline (**Table 1**). The thoracic computed tomography (CT) result suggested a nodule in the left anterior mediastinum, about 1.6 cm in diameter (**Figure 1A**). The enhanced magnetic resonance imaging (MRI) of the pituitary revealed a 3 × 2-mm nodule in the right side of the pituitary after enhancement, which was suspected as pituitary microadenoma (**Figure 1B**). All the above-mentioned tests made the identification of Cushing’s disease (CD) and EAS troublesome. A subsequent bilateral inferior petrosal sinus sampling (BIPSS) combined with desmopressin (DDAVP) stimulation test was performed, and the results showed that there was neither a lateralization ACTH rate of the bilateral inferior petrosal sinus (IPS) nor a higher ACTH level in IPS relative to peripheral blood before and after the DDAVP stimulation.

**Table 1:** Laboratory and hormone values of the patient.

Parameters	Values	Reference range
Blood potassium (mmol/L)	2.98	3.50–5.30
FBG (mmol/L)	11.22	3.90–5.90
ACTH (ng/L)	162.10	5.00–78.00
8:00 PTC (nmol/L)	2,247.00	147.30–609.30
24:00 PTC (nmol/L)	2,104.00	147.30–609.30
24-h UFC (ug/24 h)	9,174.30	20.30–127.60
ACTH after HDDST (ng/L)	222.30	
PTC after HDDST (nmol/L)	1,746.00	
24-h UFC after HDDST (ug/24 h)	3,421.20	
ACTH before DDAVP stimulation test (ng/L)	212.50	
ACTH after DDAVP stimulation test (ng/L)	1,834.00	
ACTH after the surgery (ng/L)	13.03	
PTC after the surgery (nmol/L)	224.00	

FBG, fasting blood glucose; ACTH, adrenocorticotrophic hormone; PTC, plasma total cortisol; UFC, urine-free cortisol; HDDST, high-dose dexamethasone suppression test; DDAVP, desmopressin.

**Figure 2:**
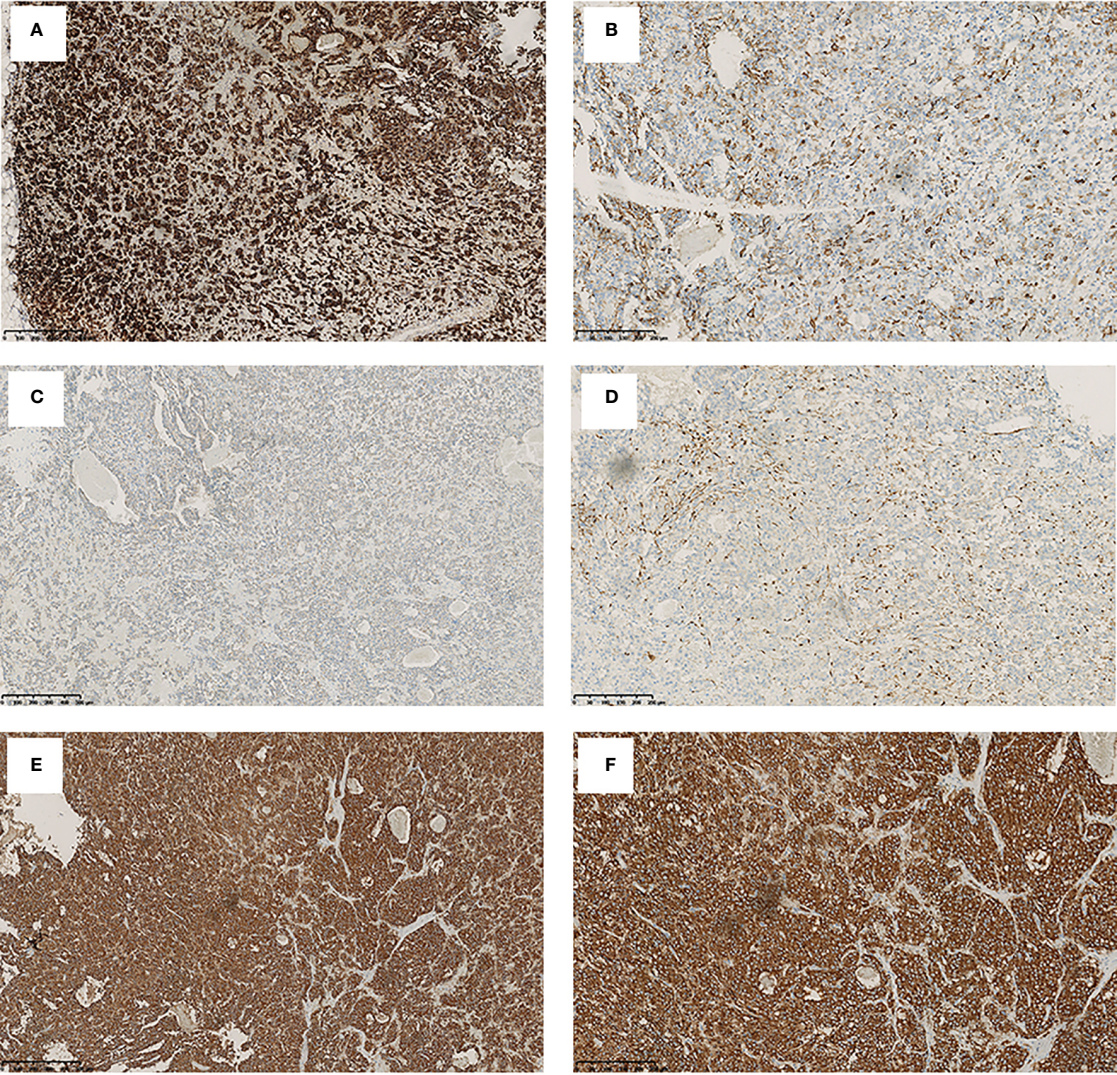
The immunohistochemical staining pattern of mediastinal mass. Tumor cells show a positivity for adrenocorticotrophic hormone **(A)** (original magnification, ×100), chromogranin A **(B)** (original magnification, ×200), PCK **(C)** (original magnification, ×100), and S100 **(D)** (original magnification, ×200) and synaptophysin **(E, F)** (original magnification, ×100 and ×200).

In order to identify the localization of the ACTH-secreting tumor, we recommended that the patient finish the positron emission tomography CT (PET/CT) scan. After the ^68^Ga-DOTATATE-PET/CT and ^18^F-fluorodeoxyglucose (FDG)-PET/CT were finished, the results showed both ^68^Ga (**Figures 1C, D**) and glucose (**Figure 1E**) uptake in the anterior mediastinal nodule, which suggested a neuroendocrine tumor. Therefore, EAS caused by the anterior mediastinal nodule was considered, and anterior mediastinal tumorectomy was performed. After the anterior mediastinal tumorectomy was performed, a 2 × 1.7 × 1-cm anterior mediastinal mass was removed. Immunohistochemical staining showed positive staining for chromogranin A and synaptophysin, which could happen in either paraganglioma or carcinoid tumors. However, S100 protein, which is a characteristic of paraganglioma, showed positive staining in the sustentacular cells. So, considering the positive staining of ACTH and PCK and the negative staining of EMA, we took the diagnosis of a paraganglioma with ACTH-secreting function (**Figure 2**).

After the operation, the ACTH and cortisol concentrations declined to low levels, with 13.03 ng/L and 224 nmol/L, respectively. The clinical symptoms were improved significantly, and the edema of both lower limbs subsided. Then, 10 mg prednisone per day was prescribed for the patient post-operation, and this dose was gradually tapered. At 6 months after the operation, the hypothalamus–pituitary–adrenal axis of the patient was recovered, and prednisone replacement therapy was stopped. He is now still presenting with total clinical remission at 16 months post-surgery. Follow-up of this patient is still ongoing (**Table 2**).

**Table 2:** Biochemical and hormone values before and after the surgery.

	Before	After	2 months later	11 months later	Reference value
PTC (nmol/L)	2,060.00	224.00	183.00	331.00	147.30–609.30
ACTH (ng/L)	330.80	13.03	33.47	31.72	5.00–78.00
Blood potassium (mmol/L)	3.04	4.79	4.67	4.50	3.50–5.30
Blood calcium (mmoI/L)	2.04	2.07	2.41	2.34	2.11–2.52
FBG (mmoI/L)	11.71	5.25	4.91	5.06	3.90–5.90

PTC, plasma total cortisol; ACTH, adrenocorticotrophic hormone; FBG, fasting blood glucose.

## Perspective of the Patient

“I was a policeman. My body was very strong before I got sick. But I became very weak since more than 1 year ago. I felt tired every day. There was no strength in my limbs, especially in the lower limbs. My mood got worse gradually, lazy words, and depression. All the symptoms progressed rapidly within 3 months, and a lumbar fracture occurred, which made me feel a terrible backache. I remember that the first time I went to the endocrinology department, I could not walk by myself and had to take a wheelchair and accompanied by my wife. After the operation, my muscle strength became better, and the mood significantly improved. At 1 to 2 months later, my symptoms gradually disappeared. Now I can come to the clinic for follow-up visits by myself, and I have resumed my routine work”.

## Discussion

Cushing’s syndrome is characterized by hypercortisolism, and its symptoms and signs are caused by long-term exposure to excessive glucocorticoids. Patients with CS clinically present with central obesity, hypertension, peripheral edema, glucose intolerance, and hypokalemia.

Cushing’s syndrome can be classified into two types: ACTH-independent and ACTH-dependent CS. In this case, the patient presented with pitting edema, full moon face, buffalo back, and elevated cortisol and ACTH concentrations. He was diagnosed with ACTH-dependent CS after initial tests. The most common cause of ACTH-dependent CS is CD, which means the endogenous secretion of ACTH from a pituitary adenoma. Only 10% of cases of ACTH-dependent CS were caused by ectopic ACTH syndrome, which stands for endogenous secretion of ACTH from the ectopic tumor (3).

The critical point and challenge of differential diagnosis for ACTH-dependent CS is to localize the ACTH-secreting tumor. In this case, the process of tumor localization was troublesome. Initially, the HDDST showed an inconsistent result in plasma and urinary cortisol. The blood ACTH and PTC were insufficiently suppressed by HDDST, whereas the 24-h urine-free cortisol was inhibited by more than 50% compared to baseline. The DDAVP stimulation test showed a positive response of ACTH. Additionally, pituitary MRI suggested an existing pituitary microadenoma with a diameter of less than 6 mm. These results made the differential diagnosis of CD or EAS controversial. Considering that the 24-h UFC concentration could be affected by urine volume and the inspection methodology, nearly 20% of ACTH-secreting pituitary microadenoma cannot be suppressed by HDDST according to previous literature (4). Previous literature had reported that some patients with ectopic ACTH syndrome could also respond to DDAVP (5). BIPSS was performed to differentiate the source of ACTH. After sampling, there was no advantage gradient of ACTH in IPS relative to peripheral blood. EAS should be highly suspected. So, ^68^Ga-DOTATATE-PET/CT and ^18^F-FDG-PET/CT were recommended to search for the ACTH source, and it was found that the anterior mediastinal nodule had increased glucose uptake and an increased expression of somatostatin receptors, which is consistent with neuroendocrine tumors that might be of ectopic ACTH source. After the anterior mediastinal mass was removed, the level of ACTH and cortisol decreased to normal range immediately. It confirmed our speculation.

This case report demonstrates that hybrid imaging modalities are of great help to improve the detection of the ectopic ACTH source, such as PET/CT, and for determination of the location and function of the tumor. The combination of ^68^Ga-DOTATATE-PET/CT and ^18^F-FDG-PET/CT could improve the detection rate of tumors, especially neuroendocrine tumors (6).

Paraganglioma originates from widely distributed specific neural crest chromaffin cells and is relatively rare in clinical practice. Paraganglioma could occur in the kidney (7), paranasal sinuses (8), and pulmonary (9, 10) and cervical (11) sites, while the mediastinum is an uncommon site of occurrence. According to the reference, the total incidence of pheochromocytomas and paragangliomas is only about 3 per million (12). Moreover, the morbidity of pheochromocytomas associated with EAS accounted for only 3% of the total EAS cases (2). Paragangliomas are classified into functional and non-functional, and functional paragangliomas mainly secrete catecholamines. ACTH-secreting paragangliomas are very rare. The postoperative pathological result reported as paraganglioma is beyond our expectation. Based on literature reviews, less than 20 cases were reported about ACTH-secreting paraganglioma (13), and only 4 cases were described to originate from the mediastinum (14). Since the patient had a clinical manifestation of neither hyper-catecholamine nor elevated serum or urinary catecholamine, a non-functional mediastinal paraganglioma secreting ACTH and leading to EAS was diagnosed eventually in this case.

Tumorectomy is the first choice for the treatment of EAS. After removal of the ectopic ACTH source, the clinical disorders of the patient can be gradually relieved. Like this patient, the edema disappeared, and the blood potassium, cortisol, and ACTH levels returned to normal after the surgery. What should be noted is that ectopic ACTH syndrome has an undesirable prognosis. The prognosis is related to the nature of the tumor. According to literature reports, only 47% of EAS patients can be cured (1). For the recrudescence of neuroendocrine tumors, even if being removed by operation, follow-up is recommended for at least 10 years (15).

## Conclusions

In summary, ectopic ACTH syndrome is challenging to locate and is easy to be missed and misdiagnosed. The dexamethasone suppression test and BIPSS lead to the correct diagnosis. Functional imaging methods with radioisotopes help localize these tumors, such as ^68^Ga-DOTATATE-PET/CT and ^18^F-FDG-PET/CT. The paraganglioma-related EAS is very rare. The optimal choice for these patients is tumorectomy. After removal of the tumor, the patient can obtain complete clinical remission. However, the evaluation of prognosis requires further long-term follow-up studies with more samples.

## Data Availability Statement

The original contributions presented in the study are included in the article/supplementary material. Further inquiries can be directed to the corresponding author.

## Ethics Statement

Written informed consent was obtained from the individual(s) for the publication of any potentially identifiable images or data included in this article.

## Author Contributions

BL took charge of original draft writing. ZY performed follow-up and management of the patient. HH contributed to manuscript review and editing. All authors contributed to the article and approved the submitted version.

## Funding

This work was supported by the Science and Technology Department of Sichuan Province (grant no. 2020YFS0193).

## Conflict of Interest

The authors declare that the research was conducted in the absence of any commercial or financial relationships that could be construed as a potential conflict of interest.

## Publisher’s Note

All claims expressed in this article are solely those of the authors and do not necessarily represent those of their affiliated organizations, or those of the publisher, the editors and the reviewers. Any product that may be evaluated in this article, or claim that may be made by its manufacturer, is not guaranteed or endorsed by the publisher.
